# Decoding the Mechanism of Shen Qi Sha Bai Decoction in Treating Acute Myeloid Leukemia Based on Network Pharmacology and Molecular Docking

**DOI:** 10.3389/fcell.2021.796757

**Published:** 2021-12-20

**Authors:** Guanfei Jia, Xiuxing Jiang, Zhiqiang Li, Xin Ding, Ling Lei, Shuangnian Xu, Ning Gao

**Affiliations:** ^1^ College of Pharmacy, Army Medical University, Chongqing, China; ^2^ Department of Hematology, Southwest Hospital, Army Medical University, Chongqing, China; ^3^ Key Laboratory of Basic Pharmacology of Ministry of Education, Joint International Research Laboratory of Ethnomedicine of Ministry of Education, Zunyi Medical University, Zunyi, China

**Keywords:** acute myeloid leukemia, network pharmacology, go, KEGG, molecular docking

## Abstract

Traditional Chinese Medicine (TCM) has been shown to be efficacious in treating leukemia for thousands of years. It has been shown that Shen Qi Sha Bai Decoction (SQSBD) has been extensively used in the treatment of acute myeloid leukemia (AML). However, the mechanism of SQSBD in treating AML remains unclear. In this study, we employed network pharmacology to analyze the potential active components and elucidate molecular mechanism of SQSBD in treating AML. A total of 268 active components were identified from SQSBD, among which 9 key components (Quercetin, luteolin, kaempferol, licochalcone A, formononetin, wogonin, β-sitosterol, oroxylin A, naringenin, and baicalein) were hit by the 6 hub targets (CDK1, MAPK1, JUN, PCNA, HSB1, STAT3) associated with leukemia. Molecular docking showed that two core active components, quercetin and licochalcone A, exhibited the highest component-like properties (DL), and could bind well to CDK1 and MAPK1 protein. The experimental validation of these two components showed that quercetin inhibited cell growth through CDK1 dephosphorylation-mediated cell cycle arrest at G2/M phase in human AML U937 and HL60 cells, and licochalcone A induced cell differentiation in these leukemia cells *via* activation of MAPK1 and upregulation of CD11b. All these results indicate that SQSBD is effective in the treatment of AML, and quercetin and licochalcone A are the major candidate compounds for AML treatment.

## Introduction

Acute myeloid leukemia (AML) is a hematologic malignant disease that is characterized by the disruption of myeloid differentiation and an abnormal proliferation of myeloid precursor cells (Newell and Cook, 2021). The incidence of AML accounts for 10–20% of leukemia in children and the 5-years survival rate for AML is only about 30% in adults (Döhner et al., 2021). Although advances in AML treatment have significantly improved the prognosis of AML patients, the 5-years survival rate accounts only 30–50%. Thus, it is critical to evaluate the molecular mechanisms of AML regulation and find novel therapeutic targets for AML treatment (Crunkhorn, 2021).

Traditional Chinese medicine (TCM) treating AML has a history of more than 2000 years in China (Chen et al., 2020). For example, TCM like arsenic trioxide exhibits therapeutic effects on AML (Balasundaram et al., 2021). The other TCM like tripterygium wilfordii has a therapeutic effect on AML (Giri et al., 2019). The China Health Commission and the Administration of Traditional Chinese Medicine issued a notice recommending formula of Shen Qi Sha Bai Decoction (SQSBD), which is composed of 12 kinds of herbal medicines including Hedyotis Diffusae Herba, Scutellariae Barbatae Herba, Lobeliae Chinensis Herba, Codonopsis Radix, licorice, Hedysarum Multijugum Maxim, Scutellariae Radix, Dioscoreaebulbiferae, Indigo Naturalis, Glehniae Radix, Asparagi Radix, and Agrimonia Eupatoria (Wang et al., 2020). As a classic Chinese medicine formula for clearing away heat and detoxification, SQSBD alone or combined with chemotherapy is widely utilized in the treatment of AML. A clinical study revealed that SQSBD combined with chemotherapy is more effective that chemotherapy alone in the treatment of AML (Jie, 2019). The total effective rate of the combination group and the chemotherapy group was 91.1 and 73.9%, respectively (Gu, 2017). However, the pharmacological mechanism of SQSBD treating AML remains largely unclear due to a lack of appropriate methods.

Recently, network pharmacology has been developed based on system biology, network biology and polypharmacology (Wang et al., 2021). This new area of research involves identifying the synergies and potential mechanisms of multi-component and multi-target drugs by analyzing various networks of complex and multi-level interactions based on omics and systems biology technologies. Since TCM prescriptions are considered as multi-component, multi-target therapies, which may meet the need to treat multiple complex diseases in an integrated manner, network pharmacological approaches are suitable for investigation of TCM on treatment of AML (Liang et al., 2021). In this study, we explored network pharmacology to investigate the therapeutic effect on AML. The goals of this study are to identify the major constituents of SQSBD, to predict putative targets of SQSBD, and to elucidate the pharmacological mechanism how SQSBD exerts antileukemic activity.

## Materials and Methods

### Cell Line, Reagents, and Antibodies

AML cell lines HL-60 and U937 were purchased from American Type Culture Collection (ATCC, Manassas, VA). Cells were cultured in RPMI-1640 medium supplemented with 10% FBS. CDK1 Rabbit pAb (310007), Phospho-CDK1 (Tyr15) Rabbit pAb (310063), ERK1/2 Rabbit pAb (343830) were purchased from Zen-bio (Chengdu, China); GAPDH (2118) and p-ERK1/2 Rabbit mAb (4376S) antibodies were purchased from Cell Signaling Technology (Boston, MA, United States). Licochalcone A (A0558), Quercetin (A0083) were purchased from Must BioTechnology (Chengdu, China). Cyclin A (751), Cyclin B1(752) were from Santa Cruz Biotechnology (Dallas, United States). Secondary Goat-anti Mouse (0741802) and Goat-anti Rabbit (0741516) antibodies were purchased from Kirkegaard and Perry Laboratories (KPL, Gaithersburg, MD, United States).

### Cell Cycle Analysis

Cell cycle analysis was performed on the harvested cell pellets fixed in Cell cycle staining fluid containing 1% Triton X-100, 38 mM Na citrate in ddH2O, and PI solution (1 mg/ml) containing RNase A (10 mg/ml). The mixture was analyzed by a flow cytometer after dark stain for 30 min. Data analysis used Flowjo software (Treestar, Ashland, OR, United States).

### Cell Differentiation Analysis

Cells were collected and resuspended in PBS containing PE-labeled anti-F4/80 (eBioscience, San Diego, CA, United States, catalog No. 124801), APC-labeled anti-CD11b (Biolegend, San Diego, CA, United States, catalog No. 101212), or CD133/1(AC-133)-PE (Miltenyi Biotechnology Company, Cologne, Germany, catalog No. 5140414163), dark shade for 30 min, washed twice in PBS, Samples were analyzed on a FACSCaliburTM flow cytometer (BD Immunocytometry Systems, San Jose, CA, United States) Data analysis used Flowjo software(Treestar, Ashland, OR, United States).

### Western Blot Analysis

The protein samples ranging from 15 to 30 μg were separated using SDS-PAGE and transferred to PVDF membranes (162–0177, Bio-Rad). Membranes were blocked with 5% fat-free dry milk in TBS buffer (Tris-buffered saline) and incubated with antibodies at 4°C for 24 h. Protein bands were detected by incubating with horseradish peroxidase-conjugated antibodies for 1–2 h. Signal was detected by Western ECL Substrate (1,705,040, Bio-Rad).

### Targets Related to Active Components in SQSBD

Oral administration is the most common form in clinical treatment, Oral bioavailability (OB), and drug likeness (DL) are the main factors that affect the activity of the drug during oral administration. Therefore, by using “Hedyotis Diffusae Herba,” “Scutellariae Barbatae Herba,” “Lobeliae Chinensis Herba,” “Codonopsis Radix,” “Licorice,” “Hedysarum Multijugum Maxim,” “Scutellariae Radix,” “Dioscoreaebulbiferae,” “Indigo Naturalis,” “Glehniae Radix,” “Asparagi Radix,” “Agrimonia Eupatoria,” as keywords, we filtered the active components of SQSBD in the Traditional Chinese Medicine System Pharmacology (https://old.tcmsp-e.com/tcmsp.php) with the criteria OB ≥ 30% and DL ≥ 0.18. The structural information, 2D molecular structure, “PubChem CID,” and “Canonical SMILES” of the active components were obtained from PubChem2. Targets of the active components in SQSBD were also obtained from the TCMSP database. Component-target network was constructed by Cytoscape 3.8.2 software.

### Identification of Differentially Expressed Genes in GSE37307

MRNA expression datasets with accession number GSE37307 was downloaded from Gene Expression Omnibus (GEO) database (https://www.ncbi.nlm.nih.gov/geo/). It contains the expression profiles of 9 healthy subjects and 30 AML subjects. Genes with |log2FC| >  1.0 and adjusted *p* values (q values) < 0.05 were selected as DEGs. Volcano plots and heat map were constructed by using the ggplot2 package in R4.1.0 software.

### PPI Network Construction and Analysis

The PPI network was built using the BisoGenet plug-in in Cytoscape 3.8.2 software, the protein network datas came from HPRD, BIND, DIP, MINT, INTACT, BIOGRID databases integrated in Cytoscape 3.8.2 software. CytoNCA plug-in in Cytoscape 3.8.2 software was used to identify core network. Subnetwork was obtained by double median degree (DMD) values (DMD > 57), core network was filtered by degree centrality (DC) values (DC > 60), and betweenness centrality (BC) values (BC > 645) (Peng et al., 2021).

### GO and KEGG Pathway Enrichment Analysis

Gene Ontology (GO) annotation is used to define and describe the functions of gene products from three aspects, namely, biological processes (BPs), cell components (CCs), and molecular functions (MFs). The Kyoto Encyclopedia of Genes and Genomes (KEGG) is a database that integrates genome, chemistry, and system function information. In order to obtain more accurate genes functional enrichment information, GO and KEGG functional enrichment analysis results were obtained by using the clusterProfiler software package of the R4.1.0 platform, and the screening criterion was adjusted *p* < 0.05.The top 10 GO terms were presented as chord, and the top 10 KEGG signaling pathways were presented as cnetplot (Potter et al., 2021).

### R2 Database Analysis

Gene expression level between healthy people and AML, was calculated using R2 database (https://hgserver1.amc.nl/cgi-bin/r2/main.cgi) and results were presented as boxplot.

### Coexpression Analysis

Cancer Genome Atlas (https://portal.gdc.cancer.gov/) is a public online database including a large number of tumors and normal samples. R4.1.0 software was used to analyze gene coexpression.

### Active Components and Hub Targets Interaction Analysis

2D structure of each active component was downloaded from PubChem (https://pubchem.ncbi.nlm.nih.gov) in SDF format and then converted into MOL2 files using a freely available Chem3D software of Chem Office 2014. The 3D structure of protein was downloaded from Protein Data Bank (https://www.rcsb.org/). Before molecular docking, the AutoDock 4.2.6 software was used to remove ligands, structure water molecules, and add polar hydrogen atoms and charges to the protein crystal structures. The Discovery Studio software and Pymol software were used to evaluate the interaction between the targets and the active components (Üstün et al., 2021).

### Statistical Analysis

Densitometric analysis was performed using the Quantity One software (Bio-Rad) to determine the relative abundance of protein expression. All data values are represented as mean ± SD. The comparisons were performed using Student’s *t*-test. *p < 0.05, **p < 0.01, and ***p < 0.001 were regarded as significant differences.

## Results

### Potential Targets of Active Components

**FIGURE 1:**
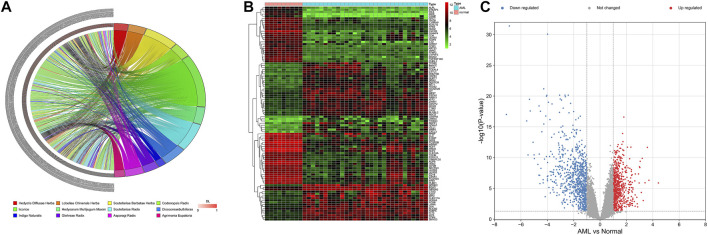
Active ingredients of SQSBD and DEGs in GSE37307. **(A)** The chord of active ingredients by using chord package. The left side of the chord is the active ingredients, arranged from top to bottom according to the size of DL, and the right side is the traditional Chinese medicine from which the active ingredients come. **(B)** The heatmap of top 100 DEGs in GSE37307 by using heatmap package. **(C)** The volcano map of DEGs in GSE37307 by using volcano package. Blue represents downregulated genes, red represents upregulated genes.

Based on TCMSP, SQSBD was derived from 12 herbal medicines including Hedyotis Diffusae Herba, Scutellariae Barbatae Herba, Lobeliae Chinensis Herba, Codonopsis Radix, licorice, Hedysarum Multijugum Maxim, Scutellariae Radix, Dioscoreae bulbiferae, Indigo Naturalis, Glehniae Radix, Asparagi Radix, and Agrimonia Eupatoria. A total of 268 active components were identified in SQSBD by using the criteria of OB 30% and DL 0.18. Detailed information of these active components is listed in Supplementary Table S1. The targets of the 268 active components were predicted by the target prediction web servers (TCMSP, BATMEN-TCM). After removing excess targets, 264 potential targets were identified with 268 active components (Supplementary Table S2). We employed R4.1.0 software to draw the chord of active components (Figure 1A). The left side of the diagram is the active components, arranged from top to bottom according to the size of DL, and the right side is the traditional Chinese medicine from which the active components come.

### Identification and Validation of DGEs in AML

We searched NCBI (https://www.ncbi.nlm.nih.gov/) for RNA sequencing data from bone marrow samples in 30 AML patients and 9 healthy subjects (normal controls). Differential expression analysis of RNA sequencing data GSE37307 in NCBI database showed that 1,505 differential expression genes (Supplementary Table S3) in AML were obtained. The distribution of the DEGs was visualized as hierarchical clustering heatmap plot and volcano plot (Figures 1B,C).

### Common Targets–Active Components Network

**FIGURE 2:**
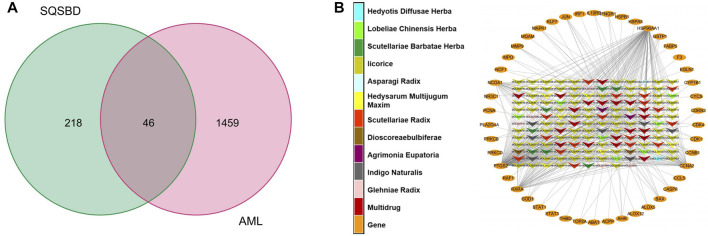
Common targets and common targets–active ingredients network. **(A)** Common targets of SQSBD and DEGs in GSE37307. **(B)** Common targets–active ingredients network. Arrow-like nodes represent the active ingredients related to the common targets, circle nodes represent common targets, the different colors of arrow-like nodes represent different traditional Chinese medicine from which the active ingredients come.

By comparing 264 component-related targets from SQSBD with 1,505 DEGs in AML, we found that AML shares 46 targets with those of the active components from SQSBD (Figure 2A). The common targets–active components network is shown in Figure 2B and Supplementary Table S4, which showed that HSP90AA1, NCOA1, PTGS2, RXRA, and CCNA2 are the most frequently targeted DEGs.

### GO Biological Process and KEGG Pathway Enrichment Analysis

**FIGURE 3:**
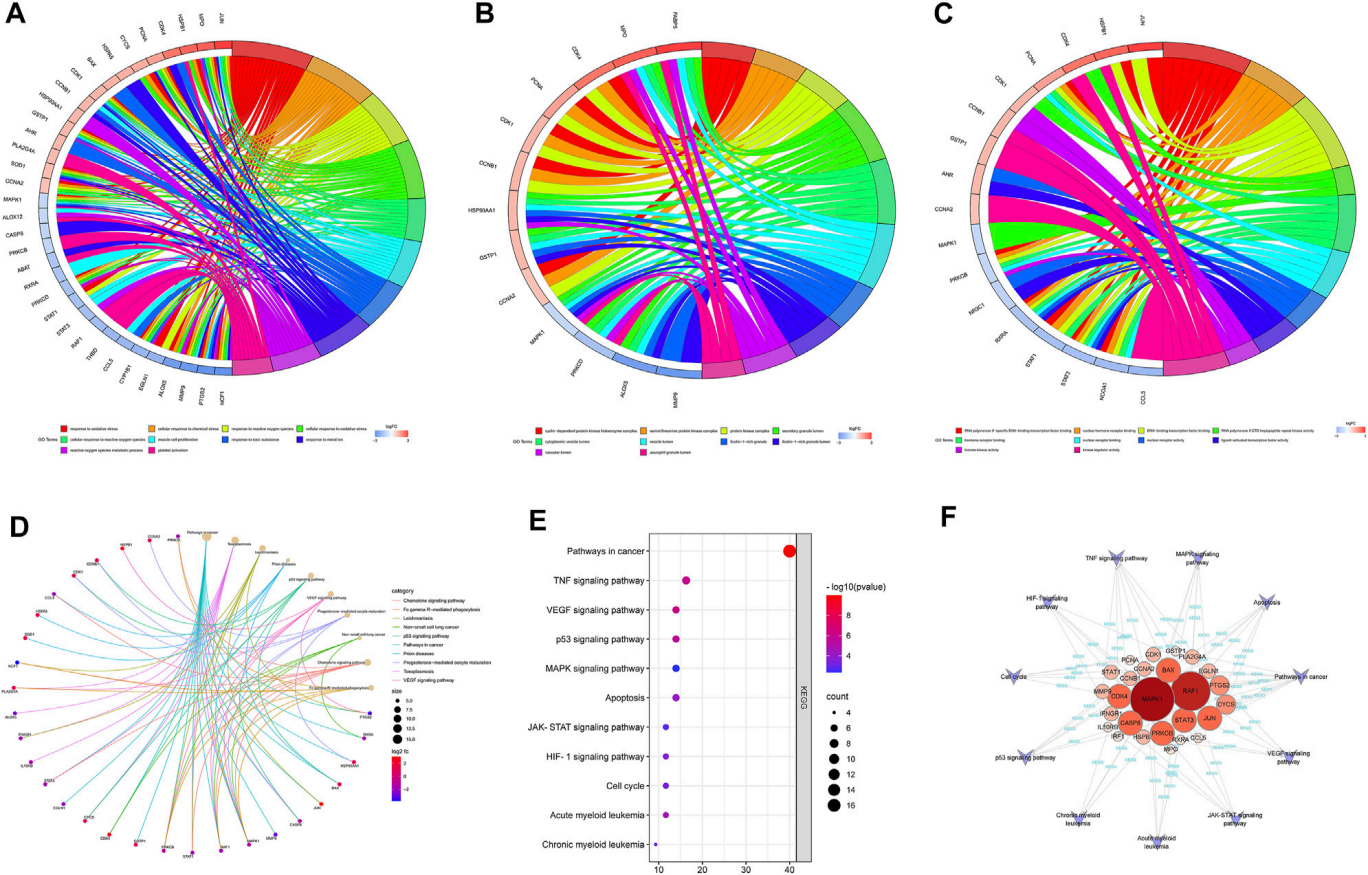
GO and KEGG analysis of Common targets. **(A)** Top 10 significantly enriched terms in biological processes (BPs) and related genes. **(B)** Top 10 signifificantly enriched terms in cellular components (CCs) and related genes. **(C)** Top 10 signifificantly enriched terms in molecular functions and related genes. **(D)** Top 10 signifificantly enriched terms in KEGG pathways and related genes. **(E)** The dot bubble of KEGG pathways related to AML. The X-axis is the number of genes and the Y-axis is the name of the terms. The darker the color, the smaller the *p* value. The larger the circle, the Ggreater the number of the target genes in the term. **(F)** Genes-KEGG network showing the KEGG pathways related to AML.

To further elucidate the mechanism of SQSBD treating AML, we performed KEGG and GO functional enrichment analysis on the 46 common targets by using the ClusterProfiler software package of the R platform. We obtained 785 BPs, 32 MFs, 63 CCs, and 109 KEGGs (*p* < 0.05) (Supplementary Table S5). The top 10 significant terms in BPs, MFs, and CCs and KEGG pathways are shown in Figure 3.

By using GO enrichment analysis, we found that BPs of the common targets are mainly associated to cellular response to oxidative stress and chemical stress, etc. (Figure 3A). The main CCs of common targets are cyclin-dependent protein kinase holoenzyme complex, serine/threonine protein kinase complex, protein kinase complex, secretory granule lumen, cytoplasmic vesicle lumen, etc (Figure 3B). The main MFs of common targets are mainly involved in RNA polymerase Ⅱ-specific DNA-binding transcription factor binding, nuclear hormone receptor binding, DNA-binding transcription factor binding, RNA polymerase Ⅱ CTD heptapeptide repeat kinase activity, hormone receptor binding, etc. (Figure 3C).

According to the KEGG pathway analysis, the critical pathways (e.g., p53, VEGF) were mainly enriched in pathways in cancer (Figure 3D). We screened out 11 KEGG pathways that were related to AML and constructed the KEGG-targets network (Figures 3E,F). The network showed that 26 targets enriched in these pathways, MAPK1 and RAF1 are the targets of most enriched signaling pathways.

### PPI Network Analysis of AML Targets

**FIGURE 4:**
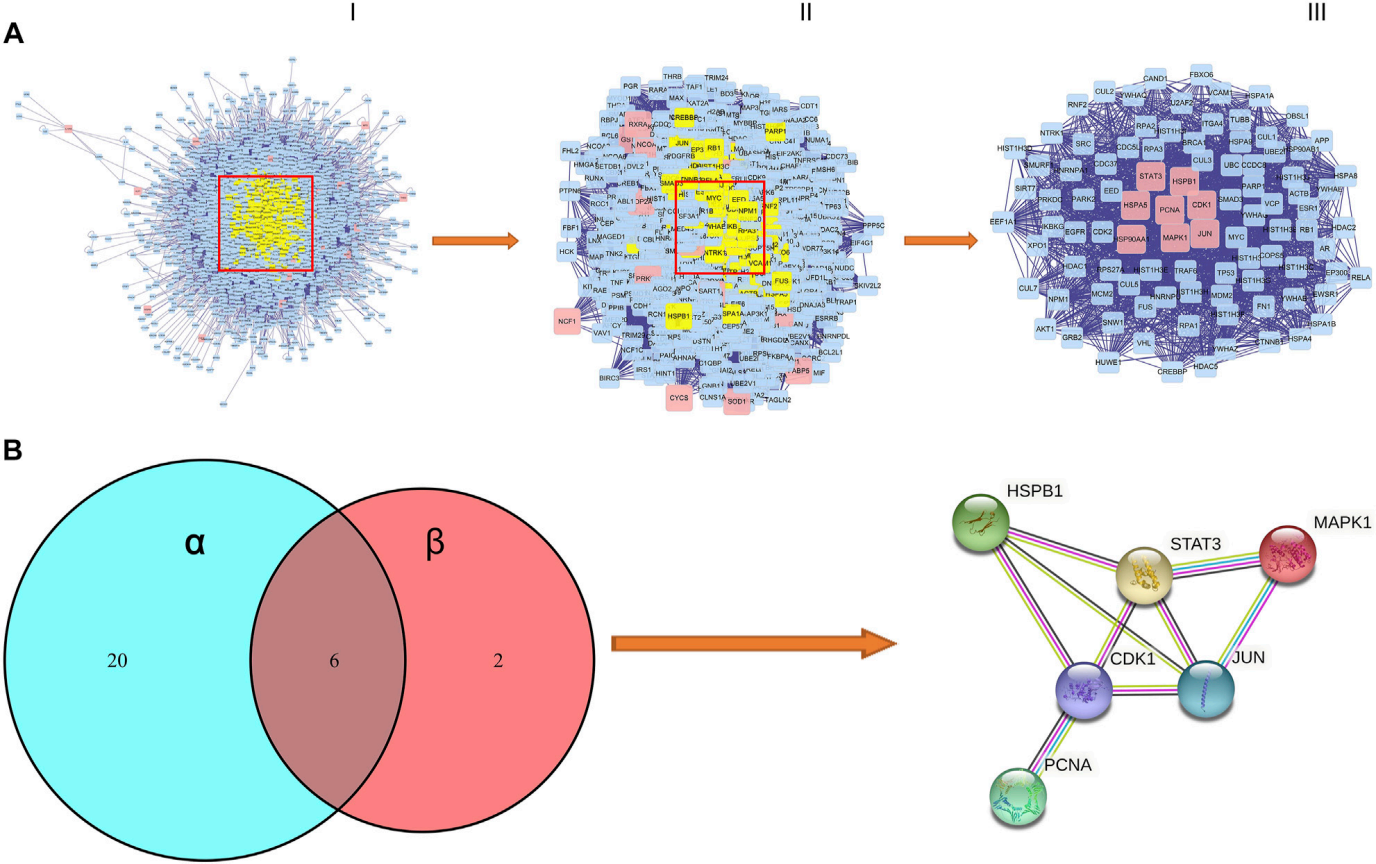
The process of topological screening for the PPI network and identifification of hub targets of SQSBD for AML. **(A)** The PPI network diagram of 96 core targets is obtained by screening 3,345 composite targets through DC, BC, CC, and 8 core targets are related to SQSBD. **(B)** Hub targets of SQSBD for AML. α represent 26 targets of KEGG pathways related to AML, β represent 8 genes of core target related to SQSBD.

We performed the protein–protein interaction (PPI) network analysis to evaluate AML targets by using Bisogenet software, and the results are shown in Figure 4A; Supplementary Table S6. The yellow nodes in the middle represent the 46 genes with highest degree values that play critical roles in the progression of AML. We then used the Cytoscape 3.8.2 software to cluster the PPI network of AML targets. We first got a rudimentary network of 3,345 nodes and 75414 edges (Figure 4A-Ⅰ). The node represents the protein, and the edge represents the interaction between proteins. By the median value of degree worth double, we then filtered a subnetwork of 893 nodes and 34741 edges (Figure 4A-Ⅱ). By using Cyto NCA software, we analyzed the network topology of the subnetwork, and had a core network of 96 nodes and 1764 edges (Figure 4A-Ⅲ). In this core network, we found that 8 core active targets were regulated by SQSBD. They are STAT3, HSPB1, HSPA5, PCNA, CDK1, HSP90AA1, MAPK1, and JUN.

We further compared 8 core active targets of SQSBD and 26 targets related to AML signaling pathways, and 6 hub targets (CDK1, HSPB1, JUN, STAT3, PCNA, and MAPK1) were obtained, these targets may play a crucial role in the treatment of AML with SQSBD, because they were involved in PPI core network, enriched in KEGG signaling pathways, and regulated by active components of SQSBD (Figure 4B).

### Role of Hub Targets in the AML

**FIGURE 5:**
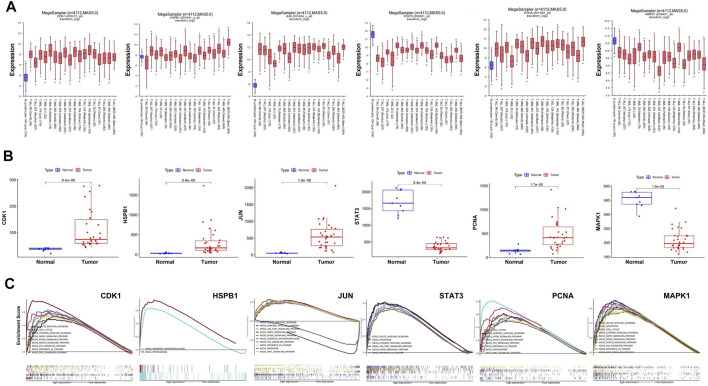
Stable expression and role of hub targets in AML. **(A)** Boxplot represents the expression level of hub targets in AML (*n* = 3,958) and normal tissues (*n* = 154) derived from the R2 database. **(B)** Boxplot represents the expression level of hub in AML (*n* = 30) and normal tissues (*n* = 9) derived from the R2 database. **(C)** GSEA analysis revealed AML-related pathways in high hub targets expression AML cohorts from TCGA datastes, respectively.

We used R2 databases to detect mRNA expression of hub targets in 1 dataset of health people and 18 datasets of AML patients, a total of 4,112 samples. The expression of hub targets in different AML samples is stable (Figure 5A), and was consistent with that in GSE37307 (Figure 5B). These results indicate that the expression of hub targets in leukemia is conserved.

**FIGURE 6:**
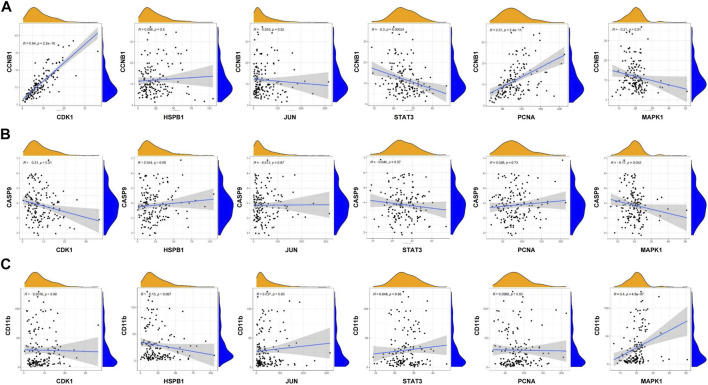
Coexpression analysis of hub targets with CCNB1, CASP9, and CD11B in AML. **(A)** Coexpression analysis of hub targets with CCNB1 revealed that there were correlation between CCNB1 and CDK1, STAT3, PCNA. **(B)** Coexpression analysis of hub targets with CASP9 revealed that the correlation between hub targets and CASP9 is not strong. **(C)** Coexpression analysis of hub targets with CD11B revealed that there is correlation between CD11B and MAPK1 (R > 0.3, *p* < 0.05).

GSEA analysis was used to conduct hub gene ontology term and pathway enrichment analysis of high- and low-expression samples. As shown in Figure 5C, the hub targets were mainly engaged in cell cycle, apoptosis and cell differentiation. Using the TCGA database, we employed CCNB1, CASP9, and CD11b as the indicators of cell cycle, apoptosis and cell differentiation, respectively, and evaluated the correlation between the hub targets and the indicators of cell cycle progression, apoptosis and cell differentiation (CCNB1, CASP9, and CD11b). The correlation analysis indicated that CCNB1 was strongly correlated with CDK1 (R = 0.84), and CD11b was correlated with MAPK1 (R = 0.4) (Figures 6A–C). Together, these results indicate that the hub targets of SQSBD, CDK1, and MAPK1, may play important roles in anti-leukemic activity through cell cycle arrest and differentiation.

### Validation by Molecular Docking

**FIGURE 7:**
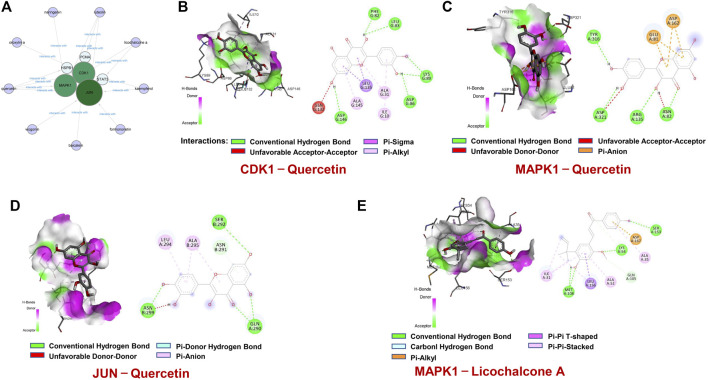
Molecular docking of active ingredients and hub targets. **(A)** The network between 6 hub targets and 9 ingredients of SQSBD. **(B)** Molecular docking of CDK1and Quercetin. **(C)** Molecular docking of MAPK1 and Quercetin. **(D)** Molecular docking of JUN and Quercetin. **(E)** Molecular docking of MAPK1 and Licochalcone A. Different colors represent the different interactions between active ingredients and the proteins.

Based on these 6 hub targets, we constructed AML-related gene targets-components network, and found that CDK1, JUN, and MAPK1 are the hub targets most enriched signal pathways regulated by 9 components of SQSBD (Figure 7A). With the criteria of OB ≥ 30% and DL ≥ 0.18, we identified 9 pivotal active components in SQSBD, namely, licochalcone A (MOL000497), quercetin (MOL000098), luteolin (MOL000006), kaempferol (MOL000422), wogonin (MOL000173), oxoxylin A (MOL002928), formononetin (MOL000392), naringenin (MOL004328), and baicalein (MOL002714), which might be the material basis for SQSBD treating AML (Table 1). Among them, licochalcone A and quercetin are the top two pivotal active components, which play important roles in the regulation of anti-leukemic activity.

**TABLE 1:** 9 pivotal active ingredients from SQSBD.

Mol ID	Molecule name	DL	AlogP	OB (%)	HL
MOL000497	Licochalcone A	0.29	4.62	40.79	16.2
MOL000098	Quercetin	0.28	1.5	46.43	14.4
MOL000006	Luteolin	0.25	2.07	36.16	15.94
MOL000422	Kaempferol	0.24	1.77	41.88	14.74
MOL000173	Wogonin	0.23	2.59	30.68	17.75
MOL002928	Oroxylin A	0.23	2.59	41.37	17.15
MOL000392	Formononetin	0.21	2.58	69.67	17.04
MOL004328	Naringenin	0.21	2.3	59.29	16.98
MOL002714	Baicalein	0.21	2.33	33.52	16.25

To confirm further the interaction between active components of SQSBD and hub targets, we used 6 hub targets and 9 pivotal active components as receptors and ligands to perform the molecular docking analysis in AutoDock vina software. For each pair of receptors and ligands, we could get 20 binding result of modes in the form of affinity, and the first mode is used as the best mode. The Discovery Studio software and Pymol software were used to evaluate the interaction between the targets and the active components, and draw 3D and 2D structure diagrams of the combined position. The −5.0 kcal/mol binding energy and 2 hydrogen bond were used as a threshold to determine whether the binding of the receptors and ligands is good or not. As shown in Table 2, all active components can bind well to the hub targets. In addition to hydrogen bond, pi–donor hydrogen bond, unfavorable donor–donor, carbon–hydrogen bond, pi–sigma, pi–cation, pi–alkyl, and alkyl may also play an important role in the combination of receptors and ligands.

**TABLE 2:** Molecular docking results between the active ingredients of SQSBD and hub targets.

Active ingredients	Hub genes	kcal/mol	Total interactions
Licochalcone A	STAT3	−6.5	11
Licochalcone A	MAPK1	−7.2	14
Quercetin	JUN	−5.4	9
Quercetin	MAPK1	−7.4	9
Quercetin	CDK1	−9.1	12
Quercetin	HSPB1	−8.9	7
Luteolin	JUN	−5.6	8
Luteolin	PCNA	−7	8
Luteolin	MAPK1	−7.5	6
Kaempferol	JUN	−5.1	5
Kaempferol	CDK1	−8.2	8
Wogonin	JUN	−5.7	7
Oroxylin A	CDK1	−8.1	7
Formononetin	JUN	−5.3	6
Naringenin	MAPK1	−7	7
Baicalein	CDK1	−8.3	4

To further verify the reliability of above molecular docking analysis, we selected top two active components of SQSBD (Quercetin and licochalcone A) as the ligands, and 3 hub targets (CDK1, JUN, and MAPK1) as the receptors, respectively. As indicated in Figures 7B–D; Supplementary Table S7, quercetin can bind well to the receptors of 3 hub targets (CDK1, JUN, and MAPK1), and had the best binding activity to CDK1 with the lowest docking score (−9.1 kcal/mol) and the highest interaction (12) (Table 2). Another component of SQSBD, licochalcone A, can only bind to the receptor of 1 hub target MAPK1 (Figure 7E), and had the best binding activity to MAPK1 with the lowest docking score (−7.2 kcal/mol) and the highest interaction (14) (Table 2). Since CDK1 is associated to the regulation of cell cycle progression and MAPK1 is related to the regulation of cell differentiation, our results indicate that SQSBD exhibits antileukemic activity. Among all the components of SQSBD, quercetin could cause cell cycle arrest at G2/M phase *via* the dephosphorylation of CDK1, and licochalcone A may induce cell differentiation *via* phosphorylation of MAPK1.

### Quercetin Causes Cell Cycle Arrest at G2/M Phase Through Inhibition of CDK1 in AML Cells

**FIGURE 8:**
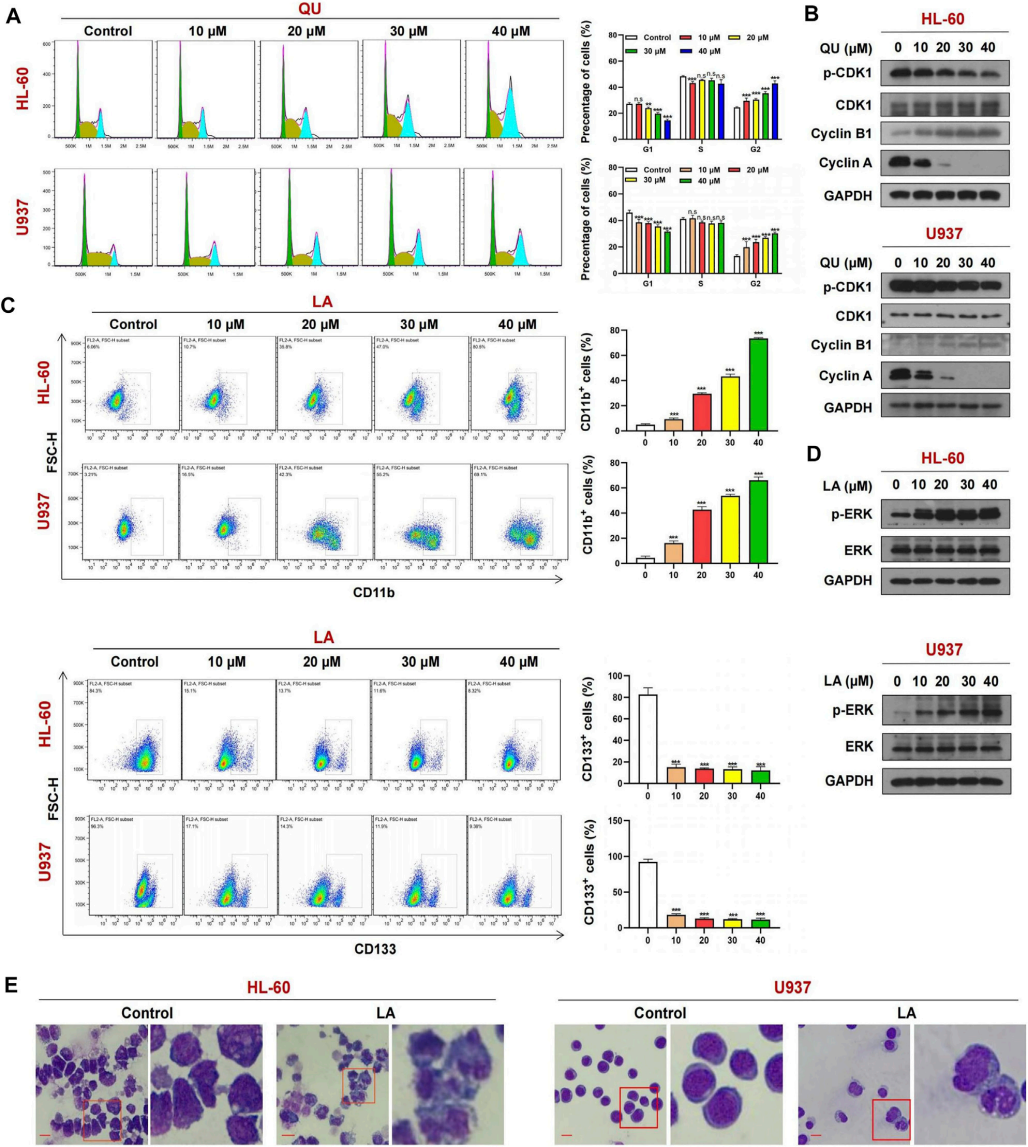
The mechanism of Quercetin or Licochalcone A in anti-AML. **(A)** Cell cycle analysis of AML cells treated with Quercetin reveals a strong increase in the G2-M cell population with a concomitant decrease in G1 population. **(B)** Cells treated with Quercetin at indicated concentrations for 24 h, whole-cell lysates were obtained and subjected to western blot analysis using antibodies against CDK1, phospho-CDK1 (p-CDK1), Cyclin A, Cyclin B1, GAPDH, **(C)** FACs analysis of CD11b and CD133 expression in AML cells lines treated with Quercetin. **(D)** Cells treated with Licochalcone A at indicated concentrations for 24 h, western blot analysis using antibodies against ERK1/2, phospho-ERK1/2 (p-ERK1/2), GAPDH. **(E)** Effects of Licochalcone A on morphology and differentiation of AML cells. Cells were treated with Licochalcone A and morphological changes were observed by phase contrast microscopy (mean ± SD, n.s, not significant, **p < 0.01, ***p < 0.001).

To further confirm whether quercetin could target CDK1, we determined the effects of quercetin on cell cycle progression in AML cells. As shown in Figure 8A, exposure of HL-60 and U937 cells to various concentrations of quercetin markedly increased the number of cells at G2/M phase in a dose-dependent manner, indicating that quercetin could cause cell cycle arrest at G2/M phase. Consistent with this result, Western blot showed that treatment of these cells with quercetin decreased the levels of phospho-CDK1, cyclin A but resulted in a markedly increase in expression of another cell cycle marker cyclin B1 in a dose-dependent manner (figure 8b). These findings indicate that quercetin causes G2/M phase cell cycle arrest through CDK1 dephosphorylation, leading to inhibition of cell proliferation in AML cells.

### Licochalcone a Induces Cell Differentiation Through Activation of MAPK1 in AML Cells

Exposure of HL-60 and U937 cells to licochalcone A at various concentrations increased the percentage of CD11b+ cells but decreased the expression of another cell differentiation marker CD133 in a dose-dependent manner (Figure 8C). Western blot indicated that treating HL-60 and U937 cells with licochalcone A increased the levels of phospho-MAPK1 (phospho-ERK) in a dose-dependent manner (Figure 8D). Wright-Giemsa staining showed that morphological features of differentiated cells were observed in licochalcone A-treated HL-60 and U937 cells, which exhibited decrease in ratio of nuclear/cytoplasmic fractions and round or leaf nuclei (Figure 8E). Together, these results suggest that licochalcone A could induce cell differentiation in AML through activation of MAPK1 signaling.

## Discussion

Accumulating evidence suggests that multiple target therapeutics is a promising strategy for drug discovery and exhibits higher efficiency and lower toxicity than monotherapies. Traditional Chinese medicines (TCMs) are characterized by a complex system of multiple components, multiple targets and multi-action pathways (Dai et al., 2021). However, the molecular mechanisms of TCMs treating diseases are hard to be elucidated because of their multicomponent nature. Network pharmacology is rapidly developing during the past few years and represents a highly attractive tool for investigating the molecular mechanism of TCMs treating diseases (Oh et al., 2021). Shen Qi Sha Bai Decoction (SQSBD) is an important multiherb remedy in TCM that has recently been shown to be effective in treating AML (Gu, 2017). However, the potential mechanism of SQSBD treating AML remains largely unknown. In the present study, we developed, for the first time, a comprehensive systems approach that combined drug target prediction, network analysis, and target validation to explore the relationships between the components in SQSBD and their putative targets and AML-related pathway systems. This study aimed to elucidate the potential mechanisms of the therapeutic effect of SQSBD in the treatment of AML by using network pharmacology approach.

According to the active components identified based on TCMSP, we performed target fishing using the TCMSP and BATMEN-TCM, obtained a total of 264 potential targets of active components from SQSBD (Zhang et al., 2021). We also used GEO database to obtaine 1,505 differential expression genes (DEGs) of AML. By comparing the potential targets of active components from SQSBD with DEGs of AML, we found that 46 potential targets of active components from SQSBD shared with DEGs of AML (Chen et al., 2021). We performed GO and KEGG enrichment analyses to further investigate the potential therapeutic targets and found that 11 signaling pathways are closely related to AML, and 26 genes are enriched on these signaling pathways (Cremer et al., 2020; Surka et al., 2021; Wang et al., 2021). In addition, by using PPI network analysis, we identified 8 core network genes which are targeted by the active components of SQSBD. By comparing 26 genes related to the signaling pathway of AML with 8 core network genes targeted by the active components of SQSBD, we obtained 6 hub targets, which are CDK1, HSPB1, JUN, STAT3, PCNA, and MAPK1. To further confirm whether these 6 hub targets are involved in the regulation of AML, we used the R2 genomic analysis to detect mRNA expression of these 6 hub targets in 18 datasets of AML patients (Hu et al., 2019). We found that these 6 hub targets were upregulated or downregulated in AML and was consistent with that in GSE37307. From GSEA analysis results, the hub targets were mainly engaged in cell cycle, apoptosis and cell differentiation (Koteluk et al., 2021). These events were confirmed by TCGA database. Such findings suggest that CDK1, HSPB1, JUN, STAT3, PCNA, and MAPK1 play important roles in the regulation of AML.

Increasing evidence reveals that molecular targets or cell signaling pathways play crucial roles in the treatment of AML through the regulation of cell cycle progression, apoptosis, cell differentiation, etc. (Bian et al., 2021). To explore the mechanism of these 6 hub targets (CDK1, HSPB1, JUN, STAT3, PCNA, and MAPK1) regulating cell cycle, apoptosis and cell differentiation, we used CCNB1 (Kantarjian et al., 2017), CASP9 (Carter et al., 2006), and CD11b (Heikamp et al., 2021) as the indicators of cell cycle, apoptosis and cell differentiation, the correlation between 6 hub targets and these indicators (CCNB1, CASP9, and CD11b) was evaluated. Among these hub targets, CDK1 was strongly correlated with CCNB1, and MAPK1 was correlated with CD11b well. Because CCNB1 is an indicator of G2/M cell cycle (Khan et al., 2021), and CD11 b is an indicator of cell differentiation (Dembitz et al., 2021), our results indicate that CDK1 and MAPK1 might play important roles in anti-leukemic activity through G2/M phase cell cycle arrest and cell differentiation.

By using TCMSP and BATMEN-TCM databases, we elucidated the chemical composition and potential mechanism of SQSBD against AML (Liu et al., 2021). A total of 268 active components were identified from SQSBD, while the targets of these components in the context of AML network were generated. By screening the components of SQSBD, 9 core active components (licochalcone A, quercetin, luteolin, kaempferol, wogonin, oxoxylylin A, formononetin, naringenin, baicalein) might be the material bases for SQSBD in the treatment of AML. Among them, licochalcone A and quercetin are the top two pivotal active components, which play important roles in the regulation of anti-leukemic activity (Talat et al., 2021). We further performed the molecular docking to confirm the interaction between active components of SQSBD and hub targets and found that licochalcone A-MAPK1 and quercetin-CDK1 had strong binding efficiencies. Because MAPK1 was positively correlated with a cell differentiation indicator CD11b, and CDK1 was positively correlated with a G2/M cell cycle indicator CCNB1, our results indicate that licochalcone A and quercetin may exhibit anti-leukemic activities through cell differentiation and G2/M cell cycle arrest.

Recent evidence reveals that MAPK1 signaling is involved in the regulation of cell differentiation (Deng et al., 2020). It has been shown that licochalcone A induces the osteogenic differentiation through activation of MAPK1 (Liu et al., 2021). Consistently, our study showed that licochalcone A induced cell differentiation with increased percentage of CD11b+ cells in a dose-dependent manner in AML U937 and HL-60 cells. This study also showed that treating U937 and HL-60 cells with licochalcone A increased the levels of phospho-MAPK1 in a dose-dependent manner. These findings suggest that a core active ingredient of SQSBD, licochalcone A, exhibits anti-leukemic activity through activation of MAPK1-mediated cell differentiation (Zhang et al., 2018).

It has been reported that quercetin inhibited cell proliferation through dephosphorylation of CDK1 mediating cell cycle arrest at G2/M phase (Bishayee et al., 2015). Similarly, our study indicated that quercetin inhibited cell proliferation through G2/M phase cell cycle arrest in a dose-dependent manner in U937 and HL-60 cells. Western blot analysis showed that treating these cells with quercetin led to decrease in levels of phospho-CDK1 in a dose-dependent manner. Because CDK1 was strongly correlated with G2/M phase cell cycle indicator CCNB1and quercetin-CDK1 had strong binding efficiency, our findings suggest that dephosphorylation of CDK1 is involved in quercetin-inhibited cell proliferation through G2/M phase cell cycle arrest.

## Conclusion

In this study, we obtained 9 pivotal active components of SQSBD in treating AML, namely, licochalcone A, quercetin, luteolin, kaempferol, wogonin, oxoxylin A, formononetin, naringenin, and baicalein. By constructing a compound–target, we screened out 6 hub targets, namely, CDK1, HSPB1, JUN, STAT3, PCNA, and MAPK1. Molecular docking showed that two core active components, quercetin and licochalcone A, exhibited the highest ingredient-like properties (DL), and could bind well to CDK1 and MAPK1 protein. The experimental validation of these two components showed that quercetin inhibited cell growth through CDK1 dephosphorylation-mediated cell cycle arrest at G2/M phase in human AML U937 and HL60 cells, and licochalcone A induced cell differentiation in these leukemia cells via activation of MAPK1 and upregulation of CD11b. All these results indicate that SQSBD is effective in the treatment of AML, and quercetin and licochalcone A are the major candidate compounds for AML treatment.

## Data Availability

The datasets presented in this study can be found in online repositories. The names of the repository/repositories and accession number(s) can be found in the article/Supplementary Material.
