# Supplementary material for: Decoding the Mechanism of Shen Qi Sha Bai Decoction in Treating Acute Myeloid Leukemia Based on Network Pharmacology and Molecular Docking
Source: Front Cell Dev Biol. 2021 Dec 20;9:796757. doi: 10.3389/fcell.2021.796757 (PMC8720969; doi:10.3389/fcell.2021.796757)
Supplement: Supplementary file 3 [file Table7.DOCX]

Table S7 Molecular docking results of hub targets and pivotal active ingredients

**MAPK1 and Licochalcone A**

**STAT3 and Licochalcone A**

**MAPK1 and Quercetin**

**CDK1 and Quercetin**

| 3D structure | 3D structure | 2D structure |
| --- | --- | --- |
| 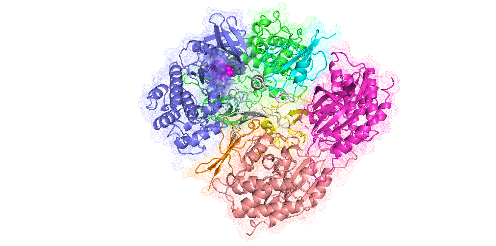 | 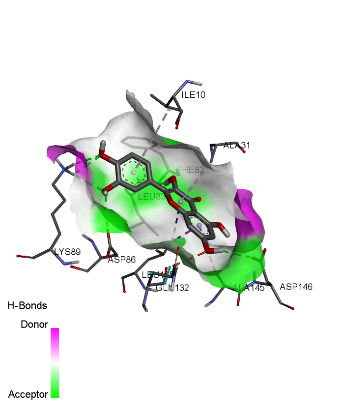 | 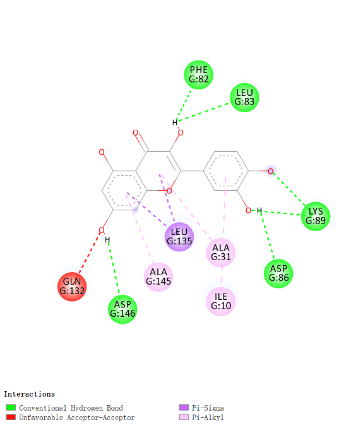 |
| 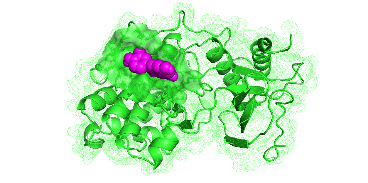 | 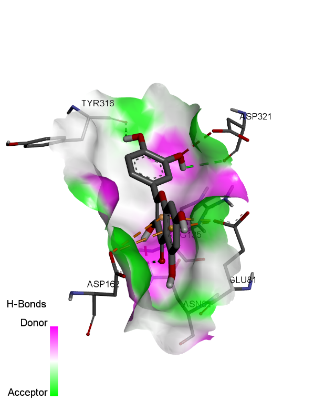 | 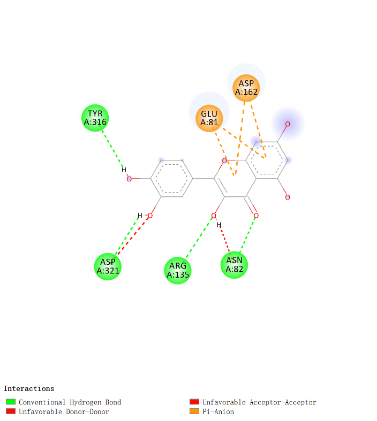 |
| 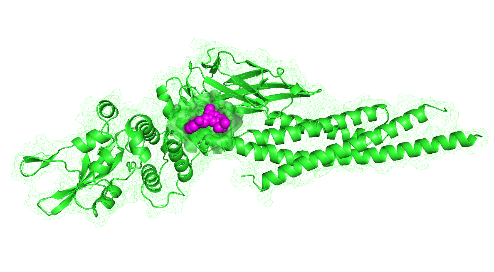 | 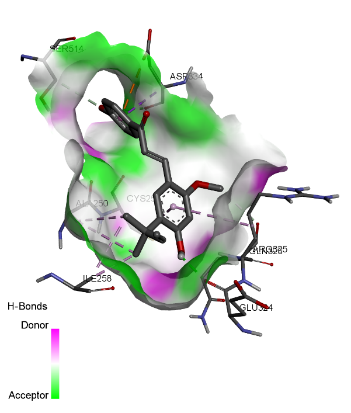 | 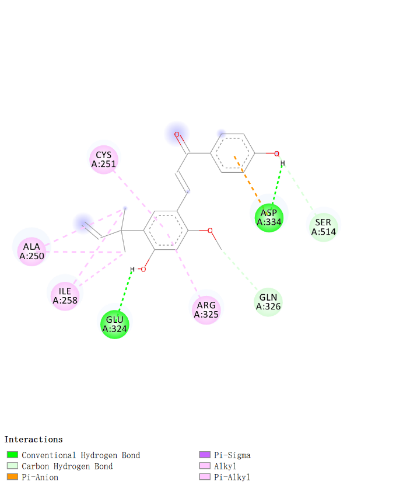 |
| 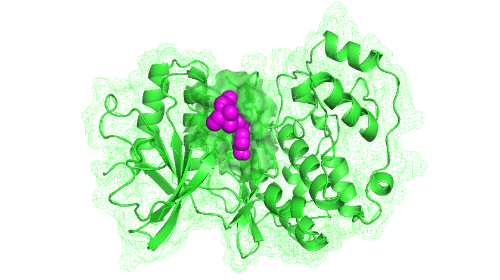 | 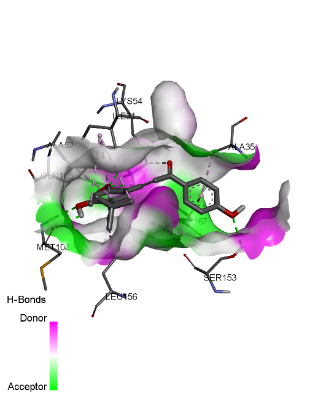 | 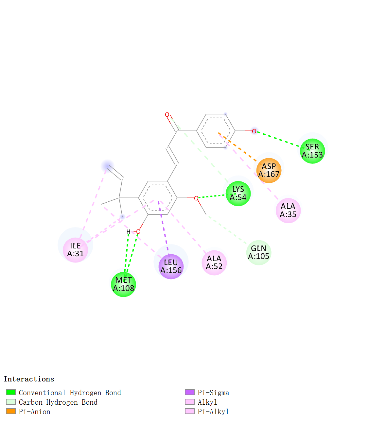 |
| 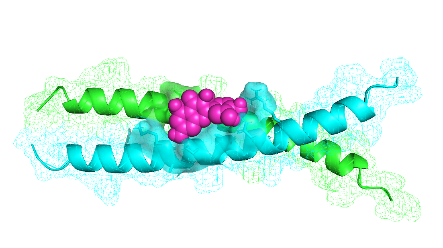  **JUN and Quercetin**  **HSPB1 and Quercetin** | 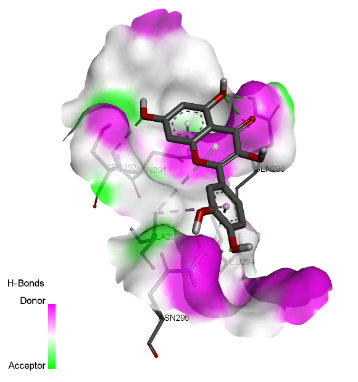 | 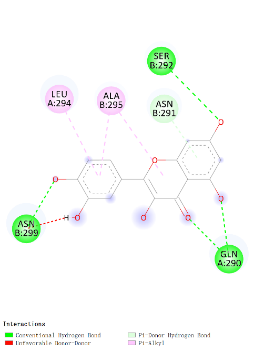 |
| 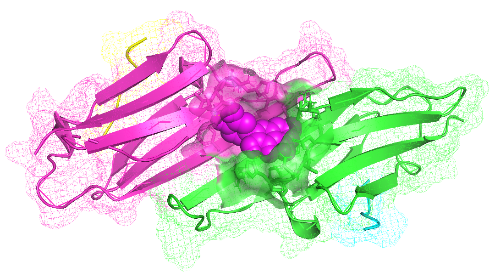  **MAPK1 and Luteolin** | 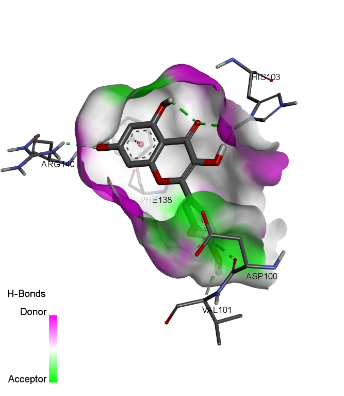 | 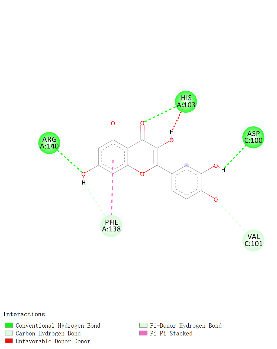 |
| 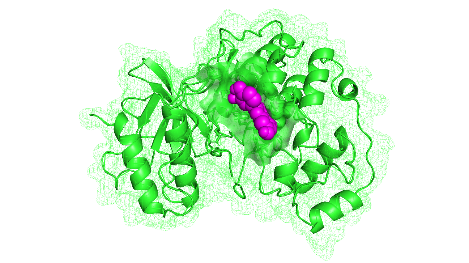  **JUN and Luteolin** | 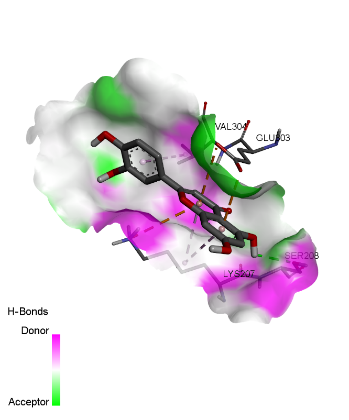 | 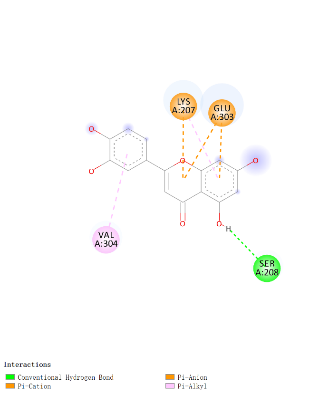 |
| 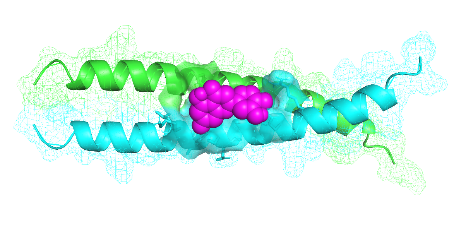 | 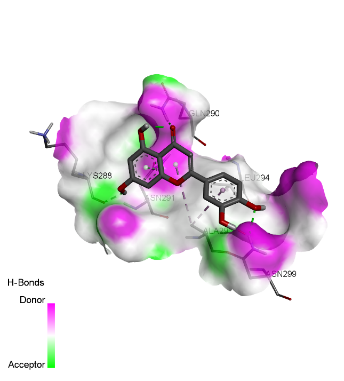 | 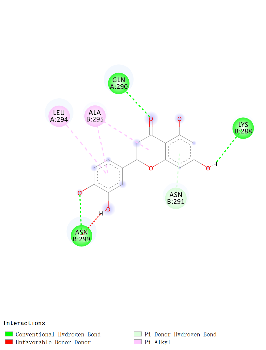 |
| 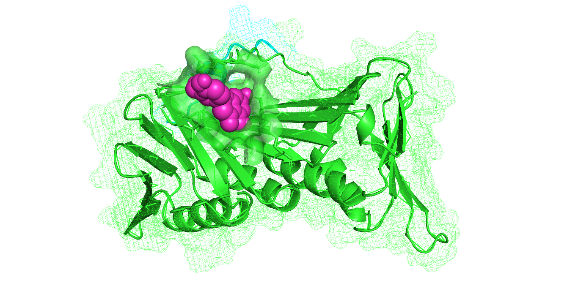  **CDK1 and Kaempferol**  **PCNA and Luteolin** | 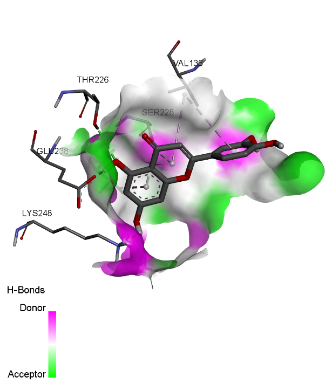 | 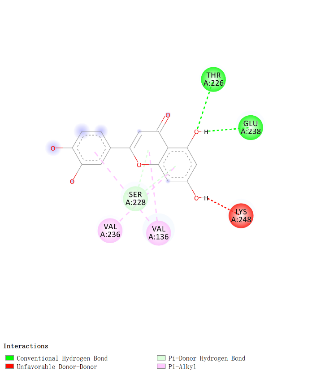 |
| 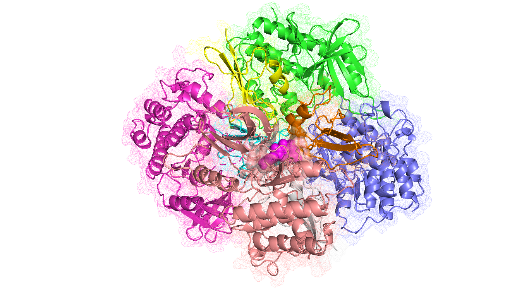  **JUN and Kaempferol** | 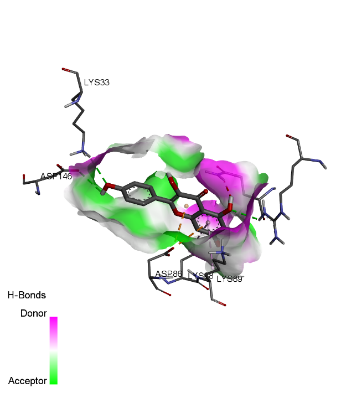 | 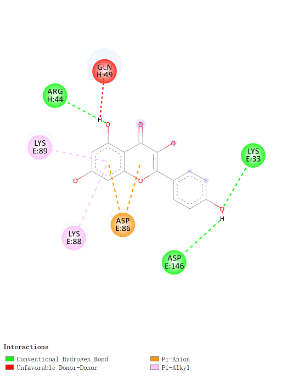 |
| 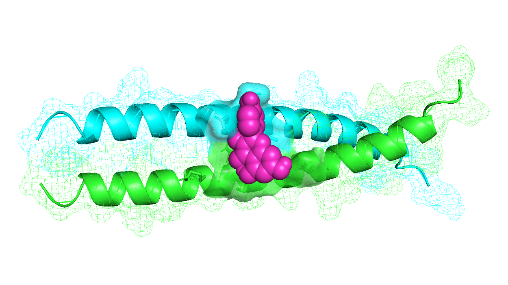  **JUN and Wogoninn** | 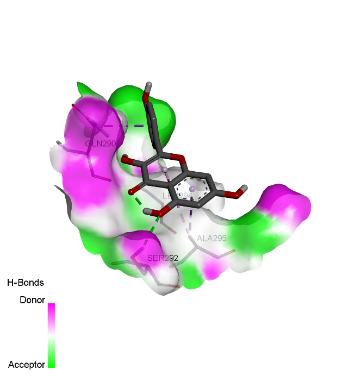 | 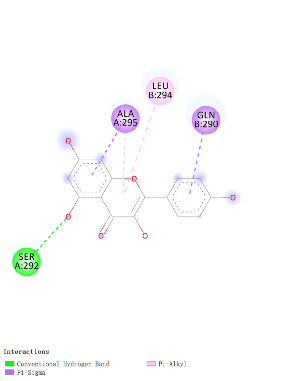 |
| 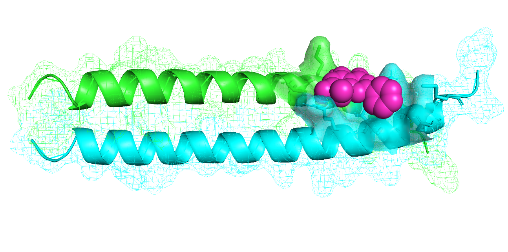 | 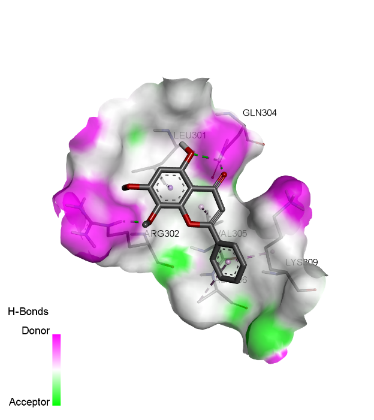 | 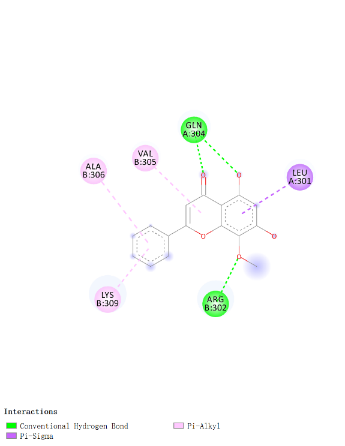 |
| 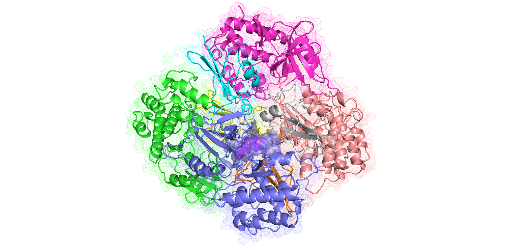 | 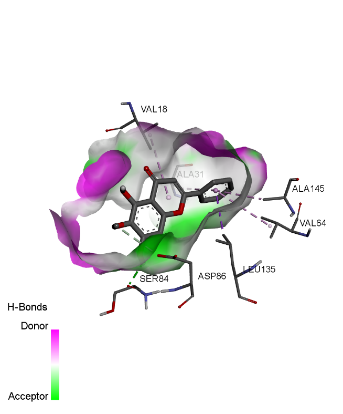 | 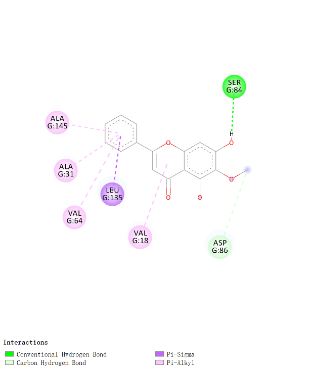 |
| 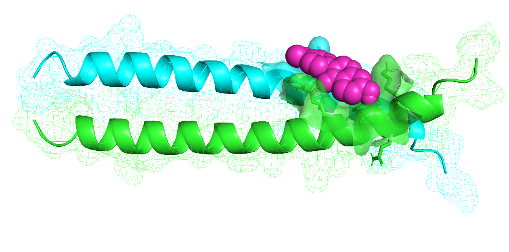 | 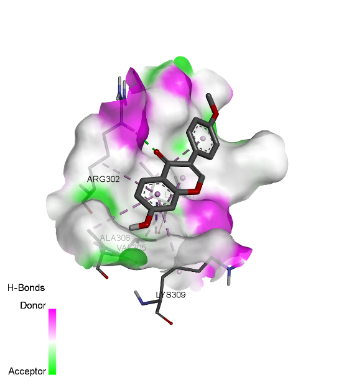 | 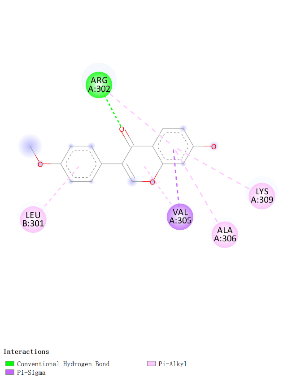 |
| 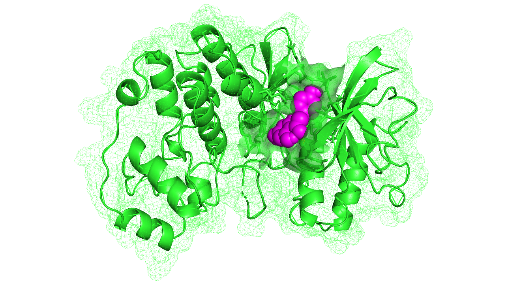 | 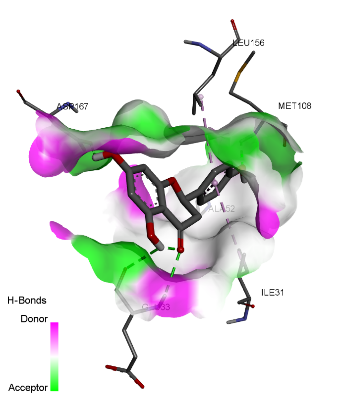 | 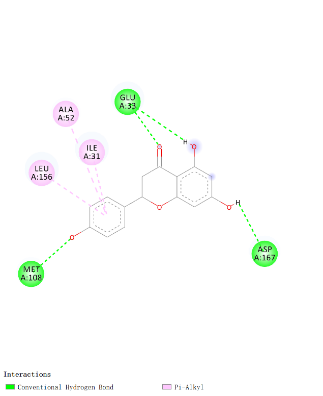 |
| 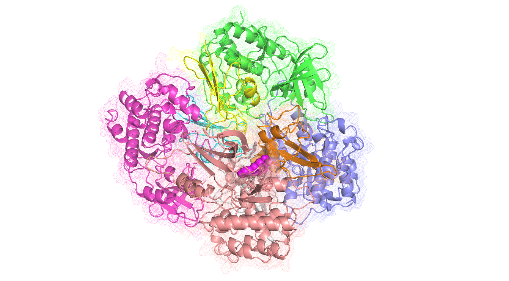 | 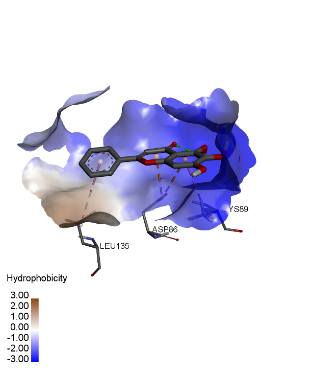 | 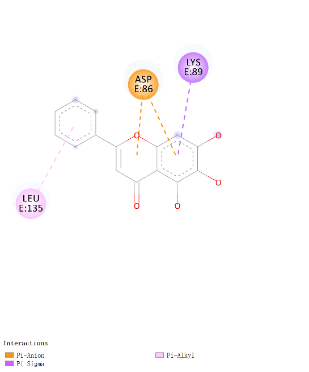 |

**MAPK1 and Naringenin**

**CDK1 and Baicalein**

**JUN and Formononetin**

**CDK1 and Oroxylin A**
